# An Information Theoretic Characterisation of Auditory Encoding

**DOI:** 10.1371/journal.pbio.0050288

**Published:** 2007-10-23

**Authors:** Tobias Overath, Rhodri Cusack, Sukhbinder Kumar, Katharina von Kriegstein, Jason D Warren, Manon Grube, Robert P Carlyon, Timothy D Griffiths

**Affiliations:** 1 Wellcome Trust Centre for Neuroimaging, Institute of Neurology, University College London, London, United Kingdom; 2 Newcastle Auditory Group, Medical School, Newcastle University, Newcastle-upon-Tyne, United Kingdom; 3 MRC Cognition and Brain Sciences Unit, University of Cambridge, Cambridge, United Kingdom; 4 Dementia Research Centre, Institute of Neurology, University College London, London, United Kingdom; McGill University, Canada

## Abstract

The entropy metric derived from information theory provides a means to quantify the amount of information transmitted in acoustic streams like speech or music. By systematically varying the entropy of pitch sequences, we sought brain areas where neural activity and energetic demands increase as a function of entropy. Such a relationship is predicted to occur in an efficient encoding mechanism that uses less computational resource when less information is present in the signal: we specifically tested the hypothesis that such a relationship is present in the planum temporale (PT). In two convergent functional MRI studies, we demonstrated this relationship in PT for encoding, while furthermore showing that a distributed fronto-parietal network for retrieval of acoustic information is independent of entropy. The results establish PT as an efficient neural engine that demands less computational resource to encode redundant signals than those with high information content.

## Author Summary

Understanding how the brain makes sense of our acoustic environment remains a major challenge. One way to describe the complexity of our acoustic environment is in terms of information entropy: acoustic signals with high entropy convey large amounts of information, whereas low entropy signifies redundancy. To investigate how the brain processes this information, we controlled the amount of entropy in the signal by using pitch sequences. Participants listened to pitch sequences with varying amounts of entropy while we measured their brain activity using functional magnetic resonance imaging (fMRI). We show that the planum temporale (PT), a region of auditory association cortex, is sensitive to the entropy in pitch sequences. In two convergent fMRI studies, activity in PT increases as the entropy in the pitch sequence increases. The results establish PT as an important “computational hub” that requires less resource to encode redundant signals than it does to encode signals with high information content.

## Abstract 3

A part of the auditory cortex (planum temporale) encodes the information content of pitch sequences.

## Introduction

We are constantly required to perceive, distinguish, and identify signals in our acoustic environment. A critical first stage of these processes is the encoding of the information into a robust neural code that allows efficient subsequent processing in the auditory system [1]. We investigated the properties of such a robust neural code at the level of the cortex by varying the amount of information—or entropy—in the acoustic signal.

In the context of information theory [2,3], entropy (*H*) denotes the uncertainty associated with an event and thus provides a metric to quantify information content: a rare—or uncertain—event carries more information than a common—or predictable—event. The properties of many information transmitting systems can be characterised in terms of entropy. Indeed, Shannon originally applied information entropy to describe transitional probabilities in language [2]: in English, less common letters (e.g., “k”) have a lower probability (or higher uncertainty) than more common letters (e.g., “e”), and therefore carry higher information and entropy. Similarly, entropy can be used to characterise pitch transition probabilities in simple musical melodies [4,5]. We used entropy to quantify the information content of pitch sequences.

**Figure 1:**
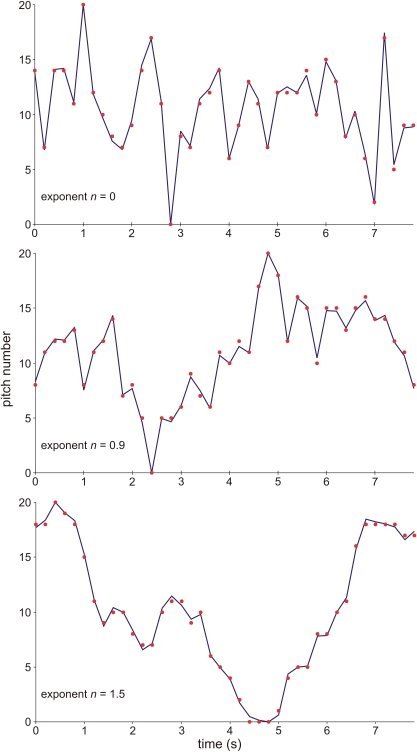
Stimuli Examples of fractal waveforms (blue) and the related pitch sequences (red, rounded to the nearest integer) based on inverse Fourier transforms of *f^–n^* power spectra, with exponent *n* = 0 (top), *n* = 0.9 (middle), *n* = 1.5 (bottom). Equitempered pitch (ten-note octave, ranging over two octaves, resulting in 21 possible pitches, with ordinal indices 0 to 21 corresponding to 300–1,200 Hz) is denoted on the *y*-axis, time (in seconds) on the *x*-axis. Entropy is largest for the top pitch sequence and decreases as exponent *n* increases.

“Fractal” pitch sequences based on inverse Fourier transforms of *f^–n^* power spectra [6,7] provide a means to control directly the entropy of the sequence via the exponent *n* (Figure 1). For *n* = 0, the excursion of the pitch sequence is equivalent to fixed-amplitude, random-phase noise and thus is completely random (high entropy). In the context of information theory, the high degree of randomness in this signal does not correspond to noise that must be removed by the system, but rather to a low predictability of the stimulus that results in each individual element of the sequence making a high degree of contribution to the information in the sequence. As *n* increases, a single stream gradually dominates the local pitch fluctuations and successive pitches become increasingly predictable (low entropy). Such stimuli are more predictable so that each element of the sequence makes little contribution to the overall information in the stimulus. These families of pitch sequences with different values of *n* are statistical “fractals” [8] in the sense that their statistical properties are scale-independent [7]. For present purposes, the critical property of these pitch sequences that we exploit here is not their fractal behaviour, but the variation of entropy that is produced as *n* varies, whilst pitch range, tempo, and pitch probability remain largely constant (however, it is inherent to the system that for large exponents *n* > 4, the pitch distribution approaches a sinusoid and consequently is tilted toward the extremes of the pitch range and also that the average interval size between successive pitches decreases for increasing exponents *n*).

Entropy for pitch sequences generated with a given value of exponent *n* can be determined by computing the sample entropy (*H*_SampEn_) [9]. Intuitively, *H*_SampEn_ is based on the conditional probability that two subsequences of length *m* that match within a tolerance of *r* standard deviations remain within a tolerance *r* of each other at the next point *m + 1*. Explicitly, for a signal or time series of length *N*, *H*_SampEn_ is defined as:

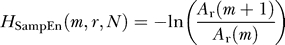
where *A*_r_(*m*) (or *A*_r_(*m + 1*)) denotes the probability that two subsequences of length *m* (or *m + 1*) match within a tolerance *r*. Two sequences “match” if their maximum absolute point-by-point difference is within a tolerance of *r* standard deviations. That is, sample entropy is essentially a measure of self-similarity, where highly self-similar time series signify high redundancy and therefore low entropy, and time series with low self-similarity represent a high degree of uncertainty and therefore high entropy. Furthermore, sample entropy is a nonparametric measure in the sense that it does not require a priori knowledge of the true probability density function of the underlying time series. In the present case, the parameters were chosen as *m* = 2, *r* = 0.5, and *N* represents the number of tones of the pitch sequence.

By varying information theoretic properties of pitch sequences, we address encoding mechanisms applied to sounds at a level of generic processing that is not specific to any semantic category. Even before such encoding mechanisms are engaged, the auditory system must represent spectrotemporal features of the stimulus in sufficient detail such that a number of different aspects of the stimulus can be encoded, in order to allow different types of subsequent categorical and semantic processing. In the current context, encoding constitutes the stage of analysis between the detailed representation of the spectrotemporal structure of the stimulus and the subsequent categorical analysis of abstracted acoustic forms. A single sound may be associated with more than one abstracted form: for example, we might obtain vowel, speaker, and position from a single sound, where each feature can undergo subsequent categorical and semantic processing. Here we use information theory to demonstrate encoding mechanisms in the brain that result in the abstraction of a form of the stimulus.

We hypothesise that if such encoding mechanisms are efficient, they will use less computational resource for stimuli that have a low information content compared with stimuli that have high information content. This hypothesis is tested by measuring the functional MRI (fMRI) blood oxygenation level–dependent (BOLD) signal as an estimate of neural activity and computational resource during the encoding of auditory stimuli in which the information content is systematically varied. We further hypothesise that processing in primary auditory cortex in the Heschl's Gyrus (HG) corresponds to a stage at which the detailed spectrotemporal structure of sounds is represented [10–12] and where such a relationship will not be observed. Instead, such a relationship is expected to be observed in distinct auditory association cortex in the planum temporale (PT), which we have previously characterised as a “computational hub” [13] that is required to convert spectrotemporal representations into “templates”—sparse symbolic neural representations that are the basis for categorical, semantic, and spatial processing. For example, the spectral envelope of a sound would represent such a template for vowel processing [14]. The model was developed to account for the involvement of PT in the analysis of a variety of complex sounds that can be processed categorically (speech, music, and environmental sounds) as well as different spatial attributes (for a review, see [13]).

Here we investigate the encoding of pitch sequences that can be like melodies in their structure, but in which the structure and information content is determined by statistical rules. We sought brain areas that display a positive relationship between the information content or entropy of pitch sequences and neural activity as assessed by the BOLD signal during encoding. Specifically, we hypothesised that such a relationship exists in PT but not in earlier auditory areas.

## Results

### Study 1

Participants were presented with pure-tone pitch sequences that were based on *f^–n^* power spectra with *n* ranging from *n* = 0–1.5 in five steps of 0.3. In a behavioural experiment before scanning, we acquired full psychometric functions demonstrating that all of the 22 participants could reliably distinguish a nonrandom pitch sequence from a random (*n* = 0) reference in a two-interval, two-alternative, forced-choice (2I2AFC) paradigm (see Materials and Methods). Perceptual thresholds for discriminating nonrandom from a random pitch sequence lay between *n* = 0.6 and *n* = 0.9 for the majority of participants.

**Figure 2:**
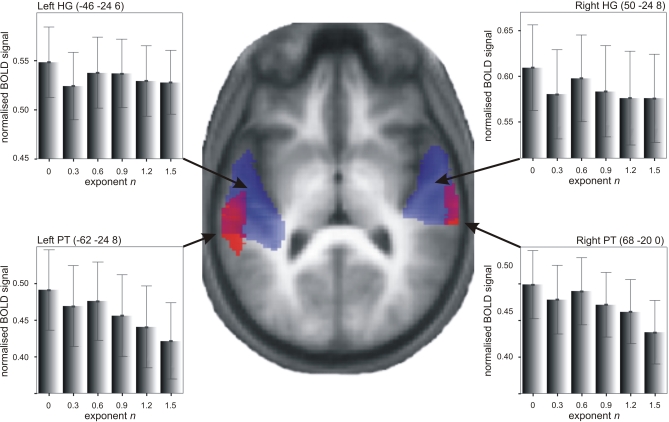
Study 1 Functional Imaging Data: Effect of Entropy Areas showing an increase in BOLD signal (*p* < 0.001, uncorrected for multiple comparisons across the brain) as a function of increasing entropy (red) and areas that responded to sound in general ([sound–silence] contrast, *p* < 0.05, FWE corrected) (blue) rendered on a tilted (pitch: −0.4) axial section of participants' normalised average structural scan. Normalised mean percent BOLD signal change (±SEM) at the local maxima in the left and right PT (bottom) and HG (top) is plotted for the six levels of exponent *n*.

In a sparse fMRI paradigm [15,16], participants listened to pitch sequences of a given value for *n* and indicated whether it was random or not. A parametric regressor based on the mean sample entropy [9] value at each of the six levels of *n* (Table 1) was used to probe for cortical areas that increased their activity with increasing entropy. The fMRI analysis revealed a BOLD signal increase in PT as a function of increasing entropy at a significance level of *p* < 0.001 (uncorrected for multiple comparisons, see Figure 2 and Table 2) and using a small volume correction for the anterior part of PT at a significance level of *p* < 0.05 (see Materials and Methods). No area increased its activity as a function of decreasing entropy, i.e., increasing predictability or redundancy.

**Table 1:**
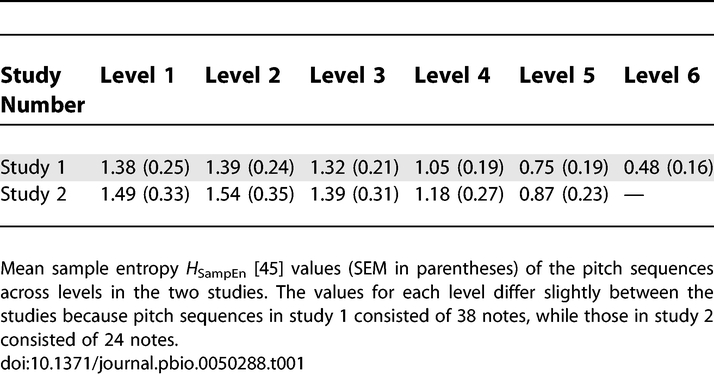
Mean Entropy Values

**Table 2:**
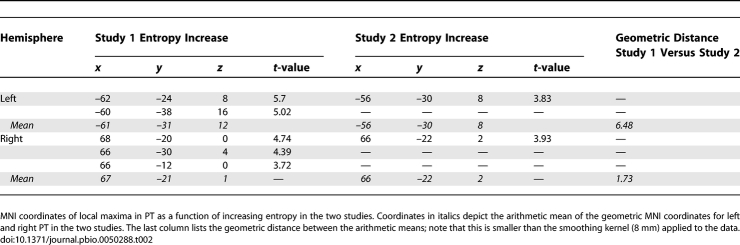
MNI Coordinates in PT

These results suggest a greater computational and energetic demand for encoding in PT as the information content of acoustic sequences (as assessed by entropy) increases. However, the present study has three potential confounds, which we addressed in a second study. First, we considered whether the effect of entropy in PT might reflect adaptation of the sensory cortical representation of frequency, as the pitch sequences were based on pure tones: for low values of exponent *n*, the frequency excursions are greater on average, so that the signal moves more between specific frequency representations, and PT might adapt less and thus produce a greater local activity. Such a mechanism would also be expected to occur in primary and secondary auditory cortex within HG. We therefore explored the specific relationship between fractal exponent and local activity in HG and PT by extracting the first eigenvariate of the BOLD signal in left and right HG as well as the local maxima in PT (see Materials and Methods). No significant difference across entropy levels was demonstrated in HG (2 Hemisphere (left, right) × 6 Entropy Level (1–6) repeated measures analysis of variance (ANOVA): no main effect of Entropy Level (*F*(5,17) = 1.11, *p* > 0.1); Figure 2). Furthermore, a 2 Area (PT, HG) × 6 Entropy Level (1–6) × 2 Hemisphere (left,right) repeated measures ANOVA demonstrated a significant difference in the relationship between BOLD signal across entropy levels in PT versus HG: Area × Entropy Level interaction (*F*(5,17) = 4.86, *p* < 0.001).

The existence of the effect in auditory association cortex in PT, the absence of an effect in HG, and a significant interaction between effects in the two areas are indirect evidence against an explanation of the results based on sensory adaptation. Nevertheless, we addressed a putative sensory explanation in a second study by using regular-interval noise, where sounds have identical passband regardless of their pitch [17–19].

Second, we also considered whether the effect of entropy might reflect perceptual adaptation at the level of the representation of pitch. Again, such an effect would not be expected in association cortex, but in a proposed “pitch centre” in lateral HG [20–22]. The second study therefore incorporated a more suitable design to detect a potential differential response to the entropy of the acoustic stimuli in cytoarchitectonic [23] and functional [20] subdivisions of HG in medial, central, and lateral HG.

Finally, we controlled for the fact that, in the first study, participants were explicitly required to assess whether the sequences were random or not. This made it possible that the results reflected a category judgment rather than a fundamental encoding mechanism. To test this, the second study differentially examined encoding and retrieval components as a function of entropy but independent of any other stimulus-related classification task.

### Study 2

**Figure 3:**
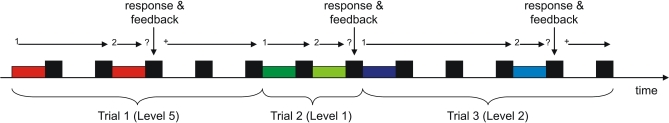
Schematic of the Experimental Design in Study 2 Depicted are three consecutive trials with pairs of pitch sequences drawn from entropy levels 5, 1, and 2. Coloured boxes indicate the presentation of a pitch sequence, black boxes indicate the acquisition of a scan volume, and the white gaps between scans denote silent periods. Identical colours within one trial indicate that the two pitch sequences of a pair are identical (trial 1), whereas slightly different hues indicate trials where the second pitch sequence was different from the first one, but drawn from the same entropy level (trials 2 and 3). There were three possible delays within and between trials: for example, trial 1 has a within-trial delay of two scans before the presentation of the second pitch sequences, and a between-trial delay of three scans before the beginning of the subsequent trial. Trial 2 has within-trial and between-trial delays of one scan, etc. Visual cues as depicted above the schematic of the design were presented to guide participants through the experiment. Participants received immediate feedback (correct/incorrect) after giving their response.

In a sparse fMRI paradigm [15,16], participants were presented with fractal pitch sequences based on *f^–n^* power spectra, with *n* ranging from *n* = 0–1.2 in four steps of 0.3. The separate pitches corresponded to regular-interval noise [17–19] (see Materials and Methods). By using broadband stimuli and an increased number of silent trials, the second study used a more suitable design to allow disambiguation of the medial functional area in HG that corresponds to the primary auditory cortex and areas in lateral HG that correspond to secondary cortices, including the area within which activity corresponds to pitch salience [20,21]. The second paradigm also enabled the disambiguation of encoding and retrieval mechanisms. Participants were scanned (1) after being required to encode a pitch sequence with a particular entropy value and (2) after listening to a second pitch sequence that was either identical to the first sequence or different from the first sequence but with the same entropy value. Activity during the first scan reflects the energetic demands of encoding the first sequence, whereas activity during the second scan reflects encoding of the second sequence, retrieval of the first, and comparison of the two. In order to decorrelate the two scans [24], we introduced a delay of one, two, or three scans between the pitch sequences (see Material and Methods and Figure 3). In contrast to the first study, participants were not informed about the nature of the pitch sequences and instead were only told that they would hear pairs of pitch sequences and that their task would be to say whether the second was same or different.

Participants' behavioural performance in the scanner was assessed via hits (*hit*) and correct rejections (*cr*) percent scores (see also Figure S2). Both mean *hit* (74.25% ± 3.14 standard error of the mean [SEM]) and mean *cr* (73.42% ± 3.31 SEM) scores were significantly above chance (50%) (one-sample *t*-test, *hit*: *t*_23_ = 7.73; *cr*: *t*_23_ = 7.08, both *p* < 0.001). Furthermore, a 2 Response (*hit*, *cr*) × 5 Entropy Level (1–5) × 3 Delay(1–3) repeated measures ANOVA showed no main effect in any of the three factors (*F*(23,1) = 0.33; *F*(20,4) = 1.1; *F*(22,2) = 0.53; all *p* > 0.05, for Response, Entropy Level and Delay, respectively). There was no Response × Entropy Level interaction (*F*(20,4) = 1.01, *p* > 0.05), indicating that participants' performance was not influenced by the entropy level of the pitch sequences. Participants had higher *cr* than *hit* scores for delay 3, whereas there were more *hits* than *cr* for delays 1 and 2 (Response × Delay interaction; *F*(22,2) = 7.91, *p* = 0.001). An Entropy Level × Delay interaction (*F*(16,8) = 2.14, *p* < 0.05) showed a performance increase for delay 1 from entropy level 1 to entropy level 5, but there was no such systematic effect for delay 2 or delay 3. There was no Response × Entropy Level × Delay interaction (*F*(16,8) = 0.45, *p* > 0.1).

**Figure 4:**
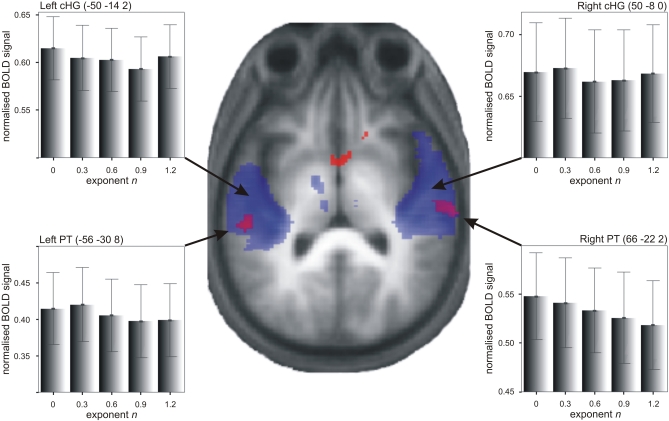
Study 2 Functional Imaging Data: Effect of Entropy Areas showing an increase in BOLD signal (*p* < 0.005, uncorrected for multiple comparisons across the brain) as a function of increasing entropy (red) and areas that responded to sound in general ([sound–silence] contrast, blue) rendered on a tilted (pitch: −0.5) axial section of participants' normalised average structural scan. Normalised mean percent BOLD signal change (±SEM) at the local maxima in the left and right PT (bottom) and central HG (cHG, top) is plotted for the five levels of exponent *n*. See Figure S1 for corresponding plots of BOLD signal in medial and lateral HG.

The imaging results replicate the findings of the first study, demonstrating that activity in PT for encoding (as assessed by both the first and second scan of each pair) increased significantly as a function of entropy for the same significance thresholds as in the first study (Figure 4 and Table 2). We examined in detail the effect at the level of primary and secondary auditory cortex by extracting the BOLD signal in medial, central, and lateral HG [20,23] (Figure 4 and Figure S1): three separate 5 Entropy Level (1–5) × 2 Hemisphere (left, right) repeated measures ANOVAs showed no main effect of Entropy Level (*F*(4,20) = 0.85, *F*(4,20) = 0.77, *F*(4,20) = 1.83, all *p* > 0.1, for medial, central, and lateral HG, respectively).

Furthermore, the relationship between entropy and BOLD signal was significantly different between PT and all three subdivisions of HG: three separate 2 Area (PT, (medial, central, or lateral) HG) × 5 Entropy Level (1–5) × 2 Hemisphere (left, right) repeated measures ANOVAs carried out for medial, central, or lateral HG showed an Area × Entropy Level interaction (*F*(4,20) = 2.61, *p* < 0.05; *F*(4,20) = 3.31, *p* < 0.05; *F*(4,20) = 5.55, *p* < 0.001, for medial, central, and lateral HG, respectively).

**Figure 5:**
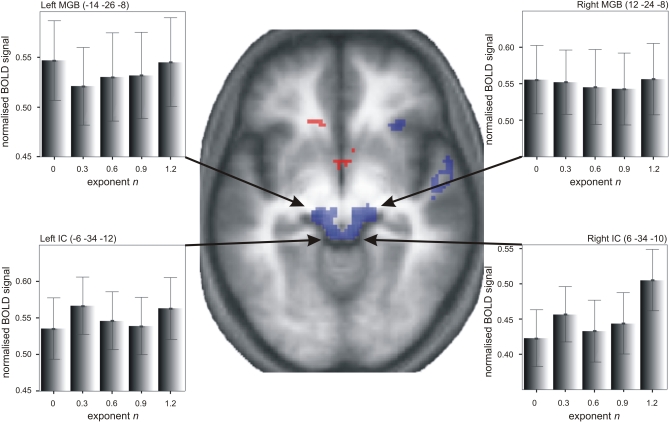
Study 2 Functional Imaging Data: Response in Subcortical Structures Entropy increase (*p* < 0.001, uncorrected) (red) and [sound–silence] (*p* < 0.05, FWE corrected) (blue) contrasts superimposed on a horizontal section (*z* = −10) of participants' normalised average structural scan that covers IC and MGB (note that *z* coordinates vary slightly between maxima and arrows, therefore they are only indicative of the exact location). Normalised mean percent BOLD signal change (±SEM) at the local maxima in left and right IC (bottom) and MGB (top) is plotted for the five levels of exponent *n*.

The cardiac gated image acquisition in Study 2 furthermore allowed an examination of a potential effect of stimulus entropy in subcortical auditory structures. We examined the relationship between entropy and the activity in the medial geniculate body (MGB) and inferior colliculus (IC) using a smaller smoothing kernel (4 mm full width at half maximum [FWHM]) that is appropriate for these subcortical structures (Figure 5). This analysis showed no main effect of entropy on the BOLD response in these areas (two separate 5 Entropy Level (1–5) × 2 Hemisphere (left, right) repeated measures ANOVAs: *F*(4,20) = 0.35, *p* > 0.1, for IC; *F*(4,20) = 1.32, *p* > 0.1, for MGB). Due to the different spatial smoothing, no meaningful interaction with the response in cortical structures can be computed.

**Figure 6:**
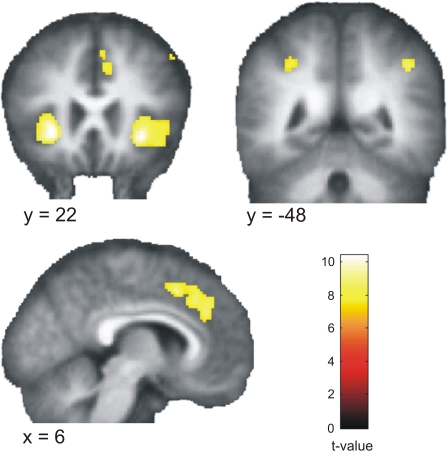
Study 2 Functional Imaging Data: Retrieval and Comparison Areas that show stronger activation (*p* < 0.05, FWE corrected) for retrieval and comparison than encoding, rendered on coronal (*y* = 22 and *y* = −48, top left and right, respectively), and sagittal (*x* = 6, bottom left) sections of participants' normalised average structural scan. See also Table S1 for exact MNI coordinates.

A second analysis based on the contrast between the second and first scans sought areas involved in retrieval and comparison, but not encoding. This contrast highlighted activity within a bilateral fronto-parietal network, including the anterior insulae and frontal opercula, inferior parietal sulci, medial superior frontal gyri, and dorsolateral prefrontal cortex (*p* < 0.05, family-wise error (FWE) corrected for multiple comparisons; Figure 6 and Table S1). A further contrast was carried out to identify an effect of entropy on retrieval and comparison, but not encoding. No effect of entropy on retrieval and comparison was demonstrated.

## Discussion

We have demonstrated an increase in the local neural activity as a function of the entropy of encoded pitch sequences in PT but not in HG. The results are consistent with a computational process in PT that requires increasing resource and energetic demands during encoding as the entropy of the sound stimulus increases.

In the first study, the use of pure tones could not exclude a possible alternate explanation of the data in terms of sensory adaptation within cortical frequency representations. The existence of the relationship in PT, but not in HG, was indirect evidence against such sensory adaptation. However, in the second study we used broadband stimuli that continually activate a broad range of cortical frequency representations irrespective of pitch, rendering explanations based on sensory adaptation untenable.

Another interpretation of these results could be based on perceptual adaptation within cortical correlates of pitch (as opposed to sensory adaptation of the stimulus representation). Previous studies have demonstrated mapping of activity within secondary auditory cortex in lateral HG as a correlate of the perceived pitch salience, whether the stimulus mapping was in the temporal domain [20] or frequency domain [21]. An explanation of the results of either study might therefore be based on adaptation within the pitch centre in lateral HG for pitch sequences with higher fractal exponent *n*. In the second study, we were able to identify separate activations in medial, central, and lateral HG. Contrary to an interpretation based on adaptation in pitch-sensitive channels, there was no relationship between the entropy and local activity in any of the subregions of HG that would have supported such an explanation. Furthermore, the interaction between HG and PT provides additional evidence for an effect of entropy that is specific to PT.

The most compelling explanation of these results is in terms of greater computational activity (and therefore local synaptic activity and BOLD signal [25]) as a function of the information content or entropy of the encoded sound. This is the first explicit demonstration of such a relationship. The results suggest an efficient form of encoding within PT, whereby sequences are encoded by a mechanism that demands less computational resource for sequences carrying low information content and high redundancy (due to the predictability of the sequence) than that required to encode sequences with little or no redundancy. “Sparse” [26–28] and “predictive” [29–31] coding both constitute such mechanisms and bases for PT acting as a computational hub [13].

In contrast, retrieval and comparison do not depend on entropy in the same way, which we propose reflects the decreased computational and energetic demands of retrieving and comparing stimuli at symbolic levels beyond stimulus encoding. The initial encoding process depends on a computationally expensive process that must abstract features from a complex spectrotemporal structure. Beyond this stage, the subsequent categorical retrieval and comparison mechanism does not depend on the detailed spectrotemporal structure. Indeed, the computational hub model [13] states that PT gates its output towards higher-order cortical areas that perform analysis at a symbolic and semantic level. We suggest that at least part of the function of PT is to compress the neural code corresponding to the initial acoustic signal (e.g., via sparse or predictive coding), and that subsequent processing is not dependent on stimulus entropy.

That PT might even perform this type of analysis in more general or supra-modal terms is suggested by work in the visual domain [32], demonstrating activation in Wernicke's area and its right-hemisphere homologue as a function of the entropy within a sequence of visually presented squares, irrespective of whether or not participants were aware of an underlying sequence. However, later studies using similar visual stimuli did not replicate this finding [33,34].

The retrieval and comparison phase highlighted a fronto-parietal network consisting of the anterior insulae and frontal opercula, inferior parietal sulci, medial superior frontal cortex, and dorsolateral prefrontal cortex. This activation pattern is common in the retrieval and comparison phase of (auditory) delayed match-to-sample tasks (e.g., [35,36]). The anterior insula in particular has been proposed as an additional auditory processing centre that allocates auditory attention, specifically with respect to sound sequences (see [37] for a review). Similarly, the parietal cortex is generally regarded as being important for attention to and binding of sensory information [38], whereas activity in the prefrontal cortex is often associated with response preparation and selection [39].

Our main aim was to study generic neural mechanisms of sound encoding as a function of entropy, and the range of pitch sequences we used included those approximating *f^−1^* (“one-over-*f*”) power spectra, which resemble many naturally occurring acoustic phenomena [40]. Notably, music and speech display *f^−1^* power spectra characteristics, reflecting the relative balance of “surprises” (e.g., musical transitions) and predictability in such signals [41,42]. Pertaining specifically to the signals used here falling in the range of *f^−1^*, two recent electrophysiological studies demonstrated preference within primary sensory cortices for *f^−1^* signals [43,44]. We did not demonstrate any “tuning” to particular values of exponent in HG (no main effect of Entropy Level; Figures 2 and 4 and Figure S1). Although we do not dismiss the possibility of neuronal preference for particular natural sequence categories at the level of HG in humans, the current studies addressed the computational and energetic demands of the perceptual encoding of sounds, rather than their sensory representation.

We have used entropy to characterise pitch sequences, but the information theoretic approach could be used to characterise sequences containing rhythm or more complex natural sound sequences. The hypothesised mechanism in PT is not a specific pitch mechanism and also predicts a similar relationship between information content and the encoding of more natural stimuli. In summary, the present data implicate PT as a neural engine within which the computational and energetic demands of encoding are determined by the entropy of the acoustic signal.

## Materials and Methods

### Study 1.

*Participants.* 30 right-handed human participants (aged 18–43 y, mean age = 24.9 y; 19 females) with normal hearing and no history of audiological or neurological disorders provided written consent prior to the experiment. None of the participants was a professional musician. The experiment was approved by the Institute of Neurology Ethics Committee, London. Eight participants had to be excluded due to excessive head movements (more than 5 mm translation or 5° rotation within one session) or not meeting the psychophysical assessment criteria (see below), leaving a total of 22 participants (aged 18–40 y, mean age = 24.2 y; 12 females).

*Stimuli.* All stimuli were created digitally in the frequency domain using Matlab (http://www.mathworks.com). Stimuli were fractal sine tone sequences based on inverse Fourier transforms of *f^–n^* power spectra [6,7] for six levels of *n* (0, 0.3, 0.6, 0.9, 1.2, and 1.5), where pitch sequences ranged from totally random (*n* = 0; high entropy) to more coherent or predictable (*n* = 1.5; low entropy). By randomising the phase spectrum, each exemplar is unique while at the same time displaying the same characteristic correlational properties of a given level. The pitch range spanned two octaves from 300–1,200 Hz, with each octave split into ten discrete equidistant pitches. Pitch sequences were presented at a tempo of five notes per second, with a total duration of 7.6 s for each pitch sequence (38 notes per sequence). There were 60 exemplars for *n* = 0 and 30 exemplars for the remaining five levels of *n*.

We calculated the mean entropy for each level of exponent *n* using the sample entropy *H*_SampEn_ [9] measure, as described in the Introduction:

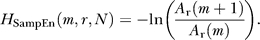



*A*_r_(*m*) denotes the probability that two subsequences of length *m* match within a tolerance *r*, i.e., *A*_r_(*m*) is the ratio of [all pairs of subsequences of length *m* that match] divided by [all possible pairs of subsequences of length *m*]; the same applies to *A*_r_(*m + 1*). Guided by Lake and colleagues [45], we chose tolerance *r* = 0.5 and length of subsequence *m* = 2 as parameter values. As Eke et al. [8] point out, taking a subset of data points from a fractal time series essentially introduces noise into the resulting time series, leading to lower *n* and consequently higher entropy estimates relative to the original values. Table 1 therefore lists the mean sample entropy values for the time series of the 38 notes in each pitch sequence.

*Experimental design.* In a behavioural experiment prior to scanning, we acquired full psychometric functions from participants discriminating the nonrandom pitch sequence against a random reference (*n* = 0) in a 2I2AFC paradigm. Participants were not given feedback. Stimuli were not the same as in the subsequent imaging paradigm and there were 72 trials (12 trials per level). Psychometric functions and 75% correct thresholds were estimated via a Weibull boot-strapping procedure [46]. Participants who did not reach at least 80% performance for levels 5 or 6 were not included in the fMRI analysis. In the functional imaging paradigm, participants were asked to categorise whether or not the pitch sequence was random by pressing the corresponding button at the end of each pitch sequence, bearing in mind that pitch sequences of intermediate levels (*n* = 0.6–0.9) are neither completely random nor completely coherent (in these cases, participants should nevertheless indicate their predominant percept). Stimuli were presented via custom-built electrostatic headphones at 70 dB sound pressure level (SPL) using Cogent software (http://www.vislab.ucl.ac.uk/Cogent/).

Gradient weighted echo planar images (EPI) were acquired with a 3-T Siemens Allegra MRI system (Erlangen, Germany), using a sparse temporal sampling technique [15,16] (time to repeat/time to echo, TR/TE = 10,530/30 ms). A total of 246 volumes (42 slices, 3 × 3 × 3 mm voxel resolution) were acquired over three sessions (82 per session), including 60 volumes for *n* = 0 and 30 volumes for the other levels of *n*, as well as 30 silent control trials (the first two volumes of each session were discarded to allow for saturation effects). To correct for geometric distortions in the EPI images due to B0 field variations, Siemens fieldmaps were acquired for each participant [47,48]. A structural T1 weighted scan was acquired for each participant [49].

*Image analysis.* Imaging data were analysed using statistical parametric mapping software (SPM2, http://www.fil.ion.ucl.ac.uk/spm). Volumes were realigned and unwarped using the fieldmap parameters, spatially normalised [50] to standard stereotactic space, and smoothed with an isotropic Gaussian kernel of 8 mm FWHM. Statistical parametric maps were generated using a finite impulse response (FIR) box-car function in the context of the general linear model [51]. The six conditions were parametrically modulated based on the average sample entropy [9] value for each level of *n* (Table 1), statistically evaluated using a random-effects model and thresholded at *p* < 0.001 (uncorrected for multiple comparisons across the brain) for areas where we had an a priori hypothesis, i.e., in auditory cortex and specifically in PT. In addition, we carried out a volume-of-interest analysis controlling for multiple comparisons within PT by centering a sphere with 1-cm radius around the centroid of the triangular anterior part of PT that is situated within the superior temporal plane as opposed to the more posterior part that abuts the parietal lobe (Montreal Neurological Institute (MNI) [*x*, *y*, *z*] coordinates [–56, –28, 6] and [58, –24, 8] for left and right PT, respectively). Our choice of volume was based on the identification of the anterior part of PT in the studies that suggested the computational hub model [13]. For areas that were not predicted a priori, we adopted a statistical threshold of *p* < 0.05 after FWE correction.

We investigated in detail a potential effect of adaptation in frequency bands at an earlier sensory level. Study 1 did not allow disambiguation of the three cytoarchitectonically [23] and functionally [20] distinct areas in HG, namely medial, central, and lateral HG (see Study 2 below for further discussion). Therefore, we identified single coordinates based on local maxima of a sound minus silence contrast for left [–46, −24, 6] and right [50, –24, 8] HG that are most similar to central HG as defined by references [20,23] and extracted the first eigenvariate of the BOLD signal at these coordinates (see Figure 2).

The BOLD signal was extracted using a standard procedure in SPM: the time series of a given voxel (e.g., the peak activation voxel for the entropy effect) is provided by SPM via a voxel-of-interest (VOI) routine. At the second-level statistical analysis, this results in a time series for each contrast where each data point corresponds to a participant. The routine is executed for each contrast, in the current case either six (Study 1) or five (Study 2) [Level–Silence] contrasts, resulting in a 22 × 6 or 24 × 5 matrix (22 or 24 participants, respectively), where each row corresponds to a participant and each column to a contrast. The threshold at which the BOLD signal was extracted was *p* < 0.05 (uncorrected for multiple comparisons). The values are then normalised to the maximum value.

Note that the interaction described here between the BOLD signal in HG and PT across levels assumes that the coupling between neuronal response and the haemodynamic BOLD signal is identical in the two brain regions. While we have no reason to assume the contrary, it has also not been proven that this is indeed the case.

### Study 2.

*Participants.* 30 right-handed participants (aged 20–44 y, mean age = 28.0 y; 16 females) with normal hearing and no history of audiological or neurological disorders provided written consent prior to the experiment. The experiment was approved by the Institute of Neurology Ethics Committee, London. Six participants had to be excluded because of excessive head movements (more than 5-mm translation or 5° rotation within one session), leaving a total of 24 participants (aged 20–44 y, mean age = 28.58 y; 12 females).

*Stimuli.* Similar to Study 1, pitch sequences were again based on *f^–n^* power spectra for five levels of *n* (0, 0.3, 0.6, 0.9, and 1.2). Each pitch was based on regular-interval noise [17–19] with 16 iterations. The pitch range spanned two octaves from 150–600 Hz, with each octave split into ten discrete equidistant pitches. Pitch sequences were presented at a tempo of four notes per second, with a total duration of 6 s for each pitch sequence (24 notes per sequence). The mean entropy values for each level of *n* are depicted in Table 1 and are slightly different from Study 1, because each pitch sequence had 24 notes instead of 38. There were 30 exemplars for each level of *n*, and stimuli were presented via custom-built electrostatic headphones at 70 dB SPL using Cogent software (http://www.vislab.ucl.ac.uk/Cogent/).

*Experimental design.* In a sparse imaging paradigm [15,16], participants were scanned (1) after being required to encode a pitch sequence with a particular entropy value and (2) after listening to a second pitch sequence that was either the same sequence or a different sequence from the same entropy level and indicating whether this was the same pitch sequence or different (see also Figure 3). To de-correlate [24] activations due to the first and second pitch sequence, the second pitch sequence followed the first pitch sequence either immediately in the next TR, or with two or three TR's delay (within-trial delay). Similarly, the first pitch sequence of the next pair could follow the second pitch sequence of the previous pair immediately, or with one or two TR's delay (between-trial delay). There were 20 pitch sequence pairs for each level, amounting to 100 encoding and 100 retrieval stimuli across the five levels of exponent *n*. In addition, there were a total of 100 within-trial volumes and 100 between-trial rest volumes. For each level of exponent *n*, 10 out of 20 pairs were identical, and 10 were different. Stimuli were counterbalanced between participants.

To guide participants, a “1” was displayed at the centre of the screen from the start of the first pitch sequence until the start of the second pitch sequence, when a “2” was displayed. At the end of the second pitch sequence, participants briefly saw a “?” to indicate they should now give their response as to whether they thought the second pitch sequence was the same as or different from the first pitch sequence. Participants received immediate feedback. During the rest period between trials, participants saw a fixation cross “+” at the centre of the screen and were instructed to relax.

Gradient-weighted EPIs were acquired with a 3-T Siemens Allegra MRI system (Erlangen, Germany), using a sparse temporal sampling technique [15,16], where each volume was cardiac gated to reduce motion artefacts (TR/TE = ∼8,800/30 ms). A total of 404 volumes (42 slices, 3 × 3 × 3 mm voxel resolution) were acquired over two sessions (the first two volumes of each session were discarded to allow for saturation effects). Subsequent to the functional paradigm, a structural T1 weighted scan was acquired for each participant [49].

*Image analysis.* Imaging data were analysed using statistical parametric mapping software (SPM5, http://www.fil.ion.ucl.ac.uk/spm). Volumes were realigned and unwarped, spatially normalised [50] to MNI standard stereotactic space, and smoothed with an isotropic Gaussian kernel of 8-mm FWHM. Statistical parametric maps were generated by modelling the evoked haemodynamic response to the stimuli and the delay period in the context of the general linear model [51].

To probe for an effect of entropy on encoding, a contrast was carried out to identify areas in which the BOLD signal in the first and second scans increased as a function of a parametric regressor based on the mean sample entropy value at each level (see Table 1). A second contrast investigated the effect of retrieval and comparison independent of encoding by subtracting the effect of encoding of the first stimulus only (corresponding to the first scan) from that to encoding of the second stimulus, retrieval of the first, and comparison of the two (corresponding to the second scan). A third contrast examined the effect of entropy on retrieval by subtracting [first scan entropy increase] from [second scan entropy increase]. Statistical results are based on a random-effects model and thresholded at *p* < 0.001 (uncorrected for multiple comparisons across the brain) for areas where we had an a priori prediction, i.e., PT, in addition to the same small volume correction (*p* < 0.05 corrected for multiple comparisons) as in Study 1. For areas that were not predicted a priori, we adopted a more conservative statistical threshold of *p* < 0.05 after FWE correction.

The second study was better suited to identify the three cytoarchitectonically [23] and functionally [20] distinct areas within HG based on the sound minus silence contrast because of (1) the greater number of silent trials and (2) the use of broadband stimuli. Three activations were identified in HG in either hemisphere, primarily to locate the lateral area previously implicated in perceptual pitch analysis [20,21] and to allow a comparison of the effect of entropy on activity here with that in PT (for individual coordinates see Table 2 for PT, Figure 2 for central and Figure S1 for medial and lateral HG).

Cardiac gating in Study 2 produced a reliable signal in subcortical structures IC and MGB (Figure 5). We reanalysed the data with a 4-mm FWHM smoothing kernel that is appropriate to these structures. Local maxima based on a sound minus silence contrast were identified in left IC ([–6, −34, −12]) and right IC ([6, –34, –10]) and left MGB ([–14, −26, −8]) and right MGB ([12, –24, –8]).

For further analysis considerations see Text S1, Figures S3 and S4, and Table S2.

## Supporting Information

Figure S1 Effect of Entropy in Medial and Lateral HG Normalised BOLD signal change (*y*-axis) in left and right medial (top) and lateral HG (bottom) (mHG and lHG, respectively) plotted against the five levels of exponent *n* (*x*-axis) for study 2. See Figure 4 for corresponding plots of BOLD signal in central HG.
(601 KB JPG)

Figure S2 Behavioural Data from Study 2 Hits (*hit*) and correct rejections (*cr*) are displayed for the three delay periods (1, 2, 3) across the five levels of exponent *n*. A repeated measures ANOVA showed no main effect of either Response (*hit* vs. *cr*), Delay (1, 2, 3) or Entropy Level (1–5). There was no Response × Entropy Level interaction, but there were a Response × Delay and Delay × Entropy Level interaction (for detailed statistical data, see Results section for study 2).
(851 KB JPG)

Figure S3 First Comparison of Analysis Techniques (Left) Comparison of results for study 1 when analysing the data with respect to the original six exponent *n* levels (red) or collapsing across levels 1 to 3, resulting in a total of 4 levels (blue). (Right) Comparison of results for study 2 when analysing the data with respect to the original five exponent *n* levels (red) or collapsing across levels 1 to 3, resulting in a total of 3 levels (blue). Results are thresholded at *p* < 0.001 (uncorrected) rendered on the tilted (pitch = −0.5) normalised mean structural of the 22 versus 24 participants. (See also Text S1.)
(291 KB JPG)

Figure S4 Second Comparison of Analysis Techniques Comparison of results for the three types of analyses in the two studies (study 1, left; study 2, right). Original analysis based on mean entropy value of the six levels derived from exponent *n* (red); analysis based on individual sample entropy value of each pitch sequence (analysis (a) (see Text S1), blue); analysis based on categorisation derived from entropy values irrespective of the exponent *n* value from which the stimuli were derived (analysis (b) (see Text S1), cyan). Results for study 1 are thresholded at *p* < 0.001 (uncorrected); results for study 2 are thresholded at *p* < 0.005 (red) and *p* < 0.05 (blue and cyan) and are rendered on the tilted (pitch = −0.5) normalised mean structural of the 22 versus 24 participants. (See also Text S1.)
(287 KB JPG)

Table S1 MNI Coordinates for Retrieval and Comparison Local maxima coordinates for the main effect of retrieval and comparison (contrast: [second scan–first scan]) at *p* <0.05 (FWE corrected for multiple comparisons across the brain). IPS, intraparietal sulcus; mSFG, medial superior frontal gyrus; VLPFC, ventrolateral prefrontal cortex; IFG, inferior frontal gyrus.
(19 KB XLS)

Table S2 Pitch Sequence Classification Descriptive data for the pitch sequences in the two studies with a classification scheme based on the sample entropy estimates. (See also Text S1.)
(18 KB XLS)

Text S1 Analysis Considerations (33 KB DOC)

## Abbreviations

2I2AFC: two-interval two-alternative forced-choice
BOLD: blood oxygenation level–dependent
fMRI: functional magnetic resonance imaging
FWE: family-wise error
HG: Heschl's Gyrus
IC: inferior colliculus
MGB: medial geniculate body
MNI: Montreal Neurological Institute
PT: planum temporale
SPL: sound pressure level
