# Supplementary material for: An Information Theoretic Characterisation of Auditory Encoding
Source: PLoS Biol. 2007 Oct 23;5(11):e288. doi: 10.1371/journal.pbio.0050288 (PMC2039771; doi:10.1371/journal.pbio.0050288)
Supplement: Supplementary file 1 [file pbio.0050288.sd001.doc]

Analysis considerations

For pitch sequences in Study 1 and Study 2 there is a significant effect of exponent *n* levels on the sample entropy estimates (ANOVA, F(5,204) = 197.814, and F(4,145) = 28.03, both p < 0.001, respectively). Post-hoc pairwise comparisons reveal that all levels except levels 1 to 3 are statistically different from each other (all p < 0.05). We therefore analysed the data by collapsing across levels whose sample entropy estimates are not statistically different from each other (levels 1 to 3 in Study 1 and Study 2, resulting in four and three levels, respectively). The results are very similar to the previous results for both studies, providing strong support for our original results (Figure S3).

We further analysed the data by (a) parametrically modulating each individual pitch sequence with its specific sample entropy value and (b) classifying pitch sequences according to their sample entropy values (ignoring the exponent *n* value from which they were derived). The second classification method resulted in the following descriptive data for the two studies (Table S2).

There is a significant effect of entropy level for Study 1 and Study 2 (ANOVA, F(4,205) = 825.51 and F(3,146) = 241.71, both p < 0.001, respectively), and significant pairwise comparisons between all levels (all p < 0.05). Importantly, the two methods for grouping the data into levels (i.e. with respect to exponent *n* or sample entropy) yield very similar classifications, as indicated by highly significant Spearman rank correlations for Study 1 and Study 2 (rho = 0.81, and rho = 0.64, both p < 0.001, respectively).

In the case of Study 1, the results are almost identical to those reported in the main manuscript, both when parametrically modulating each sequence with respect to its specific entropy value (analysis (a) above), as well as when parameterising using the mean sample entropy value for each of the five levels (analysis (b) above, Figure S4, left). In Study 2, the results of both re-analysis techniques are visible as a trend at a reduced significance threshold (Figure S4, right). The greater divergence between the original analysis and reanalysis of Study 2 is likely due to the variance of sample entropy estimates for short time series.
